# Tailoring Treatment in Cardiovascular Diseases: The Role of Targeted Therapies

**DOI:** 10.3390/pharmaceutics16040461

**Published:** 2024-03-26

**Authors:** Razan Dankar, Jad Wehbi, Marwan M. Refaat

**Affiliations:** 1 Department of Biochemistry and Molecular Genetics, American University of Beirut Faculty of Medicine and Medical Center, Beirut P.O. Box 11-0236, Lebanon; rd70@aub.edu.lb (R.D.); jw22@aub.edu.lb (J.W.); 2 Department of Internal Medicine, Division of Cardiology, American University of Beirut Faculty of Medicine and Medical Center, Beirut P.O. Box 11-0236, Lebanon

**Keywords:** targeted therapy, cardiovascular disease, monoclonal antibodies (mAbs), bispecific antibodies (BsAbs), nucleic acid drugs, cell therapy, nanoparticles, precision medicine

## Abstract

Cardiovascular diseases (CVDs) remain the leading cause of morbidity and mortality around the globe. To address this public health burden, innovative therapeutic agents are being developed to specifically target molecular and genetic markers. Various therapeutic modalities have been implemented, including vaccines, monoclonal or bispecific antibodies, and gene-based therapies. Such drugs precisely target the underlying disease pathophysiology, aiming at notable molecules such as lipid metabolism regulators, proinflammatory cytokines, and growth factors. This review focuses on the latest advancements in different targeted therapies. It provides an insightful overview of the current landscape of targeted cardiovascular therapies, highlighting promising strategies with potential to transform the treatment of CVDs into an era of precision medicine.

## 1. Introduction

Cardiovascular diseases are the leading causes of disability and mortality, and their prevalence is globally on the rise [1]. The total CVD prevalence has almost doubled from nearly 271 million in 1990 to around 523 million in 2019, while the number of CVD deaths increased steadily from around 12.1 million in 1990 to 18.6 million in 2019. As a result, CVDs tend to impose a significant socioeconomic burden on the population due to their significant contribution to the rising expenses of health care [2]. The etiology of CVDs is complex and includes genetic factors and other risk factors such as hyperlipidemia, hypertension, diabetes, obesity, smoking, and immobility. The underlying pathogenesis and progression of nearly all CVDs are primarily of atherosclerotic origin, leading to the development of coronary artery disease, cerebrovascular disease, thromboembolism, and peripheral vascular disease, ultimately resulting in myocardial infarction (MI), cardiac arrhythmias, or stroke. The clinical spectrum of cardiovascular diseases ranges widely, from asymptomatic conditions like silent ischemia to more classical presentations such as anginal chest pain, which is indicative of MI or focal neurological deficits seen in acute cerebrovascular accidents [3]. While there has been a noticeable decrease in the age-adjusted rate and mortality associated with MI due to diagnostic and treatment advancements, the overall risk of heart disease remains considerable. In fact, the general population still faces a significant 50% risk of developing heart disease by the age of 45, underscoring the persistent threat posed by cardiovascular conditions despite medical progress [3].

In the past decade, significant advances have been made in the treatment of coronary artery disease (CAD) [4]. Depending on the extent, severity, and clinical presentation of CAD, the current treatments include medical, surgical, or a combination of the two approaches. Although the aim of diminishing the risk of cardiovascular events is attained in a considerable proportion of treated patients, there is substantial patient-to-patient variation in efficacy and adverse effects, as well as frequent treatment discontinuation [5,6]. Additionally, the absence of regenerative properties in cardiomyocytes can lead to chronic cardiac dysfunction and heart failure following cardiac events. It has been documented that almost 50% of patients who experience their first MI die within 5 years post-treatment [7]. Thus, there is an imperative need to develop novel accessible and effective methods for treating CVDs, such as decreasing the infarct size post-MI and promoting regeneration.

Rising collaborations between various scientific fields, such as biotechnology and tissue engineering, have resulted in the development of novel therapeutic strategies, including stem cells, nanotechnology, and other innovations [4]. Such approaches can help in the prevention and management of CVDs while considering patient genetics, lifestyle, biomarkers, and disease phenotypes [8]. More efforts are being directed toward developing targeted treatments that deliver individualized and efficient therapies by directly targeting and binding to faulty genes or proteins. In fact, this novel modality is being implemented in various medical fields including the field of oncology, where multitargeted and highly selective kinase inhibitors are being used to treat advanced and treatment-resistant malignancies [9]. In the cardiology field, targeted therapies are becoming key players in the current treatment regimens of CVDs. We review several targeted CVD therapies and assess the prospects for this evolving field.

## 2. Monoclonal Antibodies (mAbs)

Several mAbs have demonstrated their efficacy in treating several CVDs. Table 1 summarizes targets of mAbs used in the cardiovascular field.

### 2.1. Lipid-Modifying Antibodies

#### 2.1.1. PSCK9-Targeted mAbs

Undoubtedly, statins have been the basis of lipid-lowering therapy for many years. However, despite extensive statin therapy, a considerable proportion of high-risk hypercholesterolemic individuals do not obtain satisfactory reductions in low-density lipoprotein cholesterol (LDL-C) to less than 70 mg/dL and continue to have a residual cholesterol risk [32]. Proprotein convertase subtilisin/kexin type 9 (PCSK9) is a highly intriguing and extensively explored therapeutic target in several lipid-lowering approaches. It is mainly produced by the liver and binds to the low-density lipoprotein receptor (LDLR) on the surface of hepatocytes, causing it to degrade, inhibiting hepatic clearance of low-density lipoprotein (LDL), and increasing plasma LDL-c levels [33]. As a result, gain-of-function mutations in PCSK9 can lead to autosomal dominant hypercholesterolemia, whereas people with loss-of-function mutations in PCSK9 have around 7-fold lower LDL-c levels.

In response to the discovered mechanism, three monoclonal antibodies against PCSK9, evolocumab, alirocumab, and bococizumab, were developed [34]. In 2015, the Food and Drug Administration (FDA) and the European Medicines Agency (EMA) approved the use of evolocumab and alirocumab. Bococizumab was discontinued after its evaluation in the SPIRE-1 and SPIRE-2 studies, which showed a noticeable decrease in its hypolipemic effect after a few months due to the formation of antibodies against the drug molecule, along with frequent injection site reactions [12]. On the other hand, clinical trials conducted on evolocumab and alirocumab demonstrated favorable outcomes. In the FOURIER trial, a total of 27,564 statin-treated patients with atherosclerotic cardiovascular disease and LDL-c levels of 70 mg per deciliter or higher were randomly assigned to receive subcutaneous injections of evolocumab or placebo [10]. After a follow-up of 2.2 years, results demonstrated that evolocumab reduced LDL cholesterol levels to a median of 30 mg per deciliter and significantly decreased the risk of cardiovascular events. Currently, evolocumab (Repatha) is given as 140 mg [140 mg/mL] subcutaneously (for example in the right thigh) every 2 weeks or as three 140 mg subcutaneous injections (420 mg) once a month.

Similarly, the ODYSSEY OUTCOMES trial studied the effect of alirocumab on cardiovascular events [11]. The trial included 18,924 patients with a history of an acute coronary syndrome (ACS) 1 to 12 months earlier, with an LDL-c level of at least 70 mg per deciliter, a non-high-density lipoprotein cholesterol level of a minimum of 100 mg per deciliter, or an apolipoprotein B level of at least 80 mg per deciliter. All patients had been receiving a high-intensity or maximum-tolerated dose of statin therapy. After giving the patients alirocumab or matching placebo every 2 weeks for a follow-up period of 2.8 years, alirocumab proved to have a decreased risk of recurrent ischemic cardiovascular events. Importantly, both trials showed similar adverse events among patients receiving the mAbs vs. placebo, with the exception of local injection-site reactions, which were higher in the mAb groups. With the use of PCSK9 inhibitors in clinical practice, some of the most common reported side effects were myalgias (27.2%), back pain (12%), nasopharyngitis (9.3%), headache (9.2%), upper respiratory tract infections (9%), flu-like symptoms (7.5%), and joint pain (7%) [34,35].

#### 2.1.2. ANGPTL3 Inhibitors

ANGPTL3 is a secreted glycoprotein principally produced in the liver. Early research conducted on ANGPTL3-deficient and ANGPTL3-overexpressing mice demonstrated that ANGPTL3 inhibits the activity of lipoprotein lipase (LPL), thus impairing the clearance of triglyceride-rich lipoproteins and increasing plasma triglyceride levels [36]. In a human-genetics sequencing study conducted with 58,335 participants, analysis showed that those who were heterozygous for ANGPTL3 loss-of-function variants had around 50% lower ANGPTL3 levels than noncarriers and a nearly 40% lower chance of coronary artery disease [37].

This remarkable impact of ANGPTL3 on lipid metabolism prompted interest in ANGPTL3 as a molecular target for preventing or treating dyslipidemia and CVDs. Preclinical studies in dyslipidemic mice and monkeys using evinacumab, a fully human mAb targeting ANGPTL3, showed that neutralization of ANGPTL3 reduces plasma triglycerides and LDL [38]. Similarly, human phase I and II clinical trials showed consistent favorable results [13,37]. These results were further confirmed in the phase III ELIPSE HoFH trial where 65 patients with homozygous familial hypercholesterolemia (HoFH), receiving stable lipid-lowering therapy, were randomized to receive an infusion of evinacumab or placebo. After 24 weeks, patients in the evinacumab group had a 47.1% relative decrease from baseline in the LDL-c level, whereas the placebo group’s LDL-c level increased by 1.9% [14]. Evinacumab, present under the trade name Evkeeza, was approved in 2021 by the FDA and EMA for use in patients >12 years old with HoFH [15].

### 2.2. Antithrombotic mAbs

The introduction of GP IIb/IIIa inhibitors has played a significant role in the daily practice and the treatment of MI. Abciximab, a Fab antibody fragment of chimeric human-mouse mAb 7E3, reversibly binds to platelet IIb/IIIa receptor sites, preventing platelet aggregation [39]. It blocks fibrinogen, von Willebrand factor, and other adhesive molecules from binding to GP IIb/IIIa receptors on activated platelets during clot formation. This dose-dependent inhibition creates an environment that resembles Glanzmann thrombasthenia, an autosomal recessive condition characterized by a natural decrease in GP IIB-IIIa receptors on the platelet surface.

Several studies have been carried out on the efficacy of using abciximab in patients with ST-elevation myocardial infarction (STEMI) undergoing percutaneous coronary intervention (PCI), and have proven its benefit as an adjunctive pharmacotherapy, with a greater degree of myocardial salvage and survival, but a higher risk of observed bleeding [16,17,18]. A recent meta-analysis was performed in 2022 to assess the short-time efficacy and safety of abciximab in 5008 patients with STEMI undergoing PCI with or without abciximab. The results revealed that abciximab could lead to a significant decrease in the risk of reinfarction, revascularization, and all-cause death at 6 months, but could increase the risk of minor bleeding and thrombocytopenia [19]. Other small-molecule GP IIb/IIIa inhibitors (eptifibatide, tirofiban) have been the subject of less research. Although they appear to have comparable outcomes with abciximab among STEMI patients undergoing primary PCI in terms of angiographic reperfusion, reinfarction, and 30-day mortality, more research is required to assess variations in other clinical endpoints [20,21].

### 2.3. Inflammation-Modulating mAbs

#### 2.3.1. IL-1 Inhibitors

It has now been conclusively proven that inflammation participates causally in CVDs and atherosclerosis and is especially important in the transition from stable to unstable plaques, which contribute to the clinical symptoms of the disease [40]. The IL-1 superfamily of cytokines and its receptors play a key role in the pathogenesis of CVDs by impairing endothelial cells’ ability to function normally, increasing oxidative stress, inducing the formation of procoagulant substances, and decreasing vasodilation, all of which can hasten atherosclerosis [41]. Several IL-1 receptor antagonists have been studied in clinical trials, summarized in Table 2.

Anakinra is the first FDA-approved recombinant human IL-1 receptor antagonist that competitively blocks IL-1 β and IL-1 α. It has a good safety profile and a short half-life of 4 to 6 h allowing better titration, but it needs to be administered daily via subcutaneous injection. This might create a problem for the patient, especially when accompanied by the drug’s injection-site reactions [42]. A previous pilot randomized controlled trial (RCT) involving 10 STEMI patients, who were randomized to receive 100 mg/day subcutaneous anakinra or placebo for 14 days in a double-blind manner, demonstrated that anakinra significantly improved left ventricular end-systolic and end-diastolic volume index, measured both by cardiac magnetic resonance imaging (CMR) and by echocardiography [23]. The results also showed a similar statistically significant difference in cardiac index changes among the groups, with less reduction in the anakinra group. However, there was no significant difference in left ventricle ejection fraction (EF), infarct size at late gadolinium enhancement, trans-mitral Doppler spectra and/or tissue Doppler spectra, and blood pressure among the two groups. Despite the significant limitation of the small sample size, the data pointed to anakinra’s advantageous effect on left ventricle remodeling [23]. In another phase II double-blind RCT, the VCUART3 trial, 99 STEMI patients were allocated to 2 weeks treatment with 100 mg anakinra once per day, 100 mg anakinra twice daily, or placebo, and revealed no difference between anakinra dose regimens, with a significant reduction in inflammation and high sensitivity CRP with the use of anakinra with no significant difference among the anakinra arms [22]. There was a similar dramatic reduction in death, heart failure incidence, death, and hospitalization for heart failure but no discernible impact of anakinra on left ventricle function [22]. Also, in the field of heart failure (HF), the recently published REDHART RCT studied the effect of anakinra vs. placebo on exercise capacity in 60 patients with recently decompensated systolic HF [24]. It revealed that IL-1 blockade improved peak aerobic exercise capacity by improving patient perceptions of dyspnea on exertion (DOE) and rating of perceived exertion (RPE). Patients receiving 12 weeks of anakinra experienced a time-dependent reduction in RPE and DOE values over time. However, there was no improvement in the main variables of cardio-pulmonary exercise testing including peak oxygen consumption (Vo2) and ventilatory efficiency (VE/Vco2 slope) in patients treated with anakinra [24].

In a similar manner, canakinumab is an approved human monoclonal antibody that targets the inflammatory pathway mediated by IL-1β. Unlike Anakinra, it has a lengthy elimination half-life of 26 days, making it particularly suitable for long-term use [43]. The CANTOS trial is a RCT that assigned 10,061 patients with MI and a high-sensitivity C-reactive protein (CRP) of 2 mg/L or more [25]. The study compared three dosages of subcutaneously administered canakinumab (50 mg, 150 mg, and 300 mg, given every three months) to placebo. After a median of 3.7 years of follow-up, canakinumab significantly decreased high-sensitivity CRP levels in comparison to baseline, but with no significant reduction from baseline in the LDL-c. Patients in the 150 mg group had a significant reduction by 15% compared to placebo in nonfatal MI, nonfatal stroke, and cardiovascular death, whereas canakinumab 50 mg and 300 mg did not demonstrate any superiority in those endpoints over placebo. In addition, the 150 mg canakinumab group had a decrease in hospitalization for urgent revascularization-requiring unstable angina by 17% compared to the placebo group [25]. These results suggest that an intermediate dose of canakinumab might provide cardiovascular protection in patients with CVDs. In the field of peripheral artery disease (PAD), a phase II RCT has indicated that canakinumab has not been shown to alter the progression of atherosclerotic plaque in the superficial femoral artery, but it can improve maximal and pain-free walking distance as early as 3 months following treatment [26]. More extensive research aimed at this endpoint will be necessary to demonstrate this conclusively.

Rilonacept is the final representative of this class of IL-1 blocking agents; it is a dimeric IL-1 recombinant receptor that binds IL-1α and IL-1β [44]. It is administered subcutaneously and has a half-life of around 26 days with the most common adverse events being injection-site reactions and infections of the upper respiratory tract. In a recent phase III RCT, which included 86 patients with recurrent pericarditis symptoms and with systemic inflammation, it was found that rilonacept could lead to a more significant reduction in pericarditis recurrence than placebo and could decrease pericarditis symptoms in recurrent episodes [27].

#### 2.3.2. IL-6 Inhibitors

IL-6 is another inflammatory marker that plays a significant role in CVD. Elevated levels of IL-6 (≥5 ng/L) have been associated with an augmented risk of coronary artery disease [40]. A phase II RCT randomized 117 patients with non-ST-elevation myocardial infarction (NSTEMI) to receive placebo or tocilizumab, an IL-6 receptor-targeting mAb, before coronary angiography [28]. Results showed that tocilizumab had no safety issues and could reduce high-sensitivity CRP and decrease troponin release, thus suggesting its ability to attenuate the inflammatory response and the cardiac injury post-NSTEMI. A subsequent clinical trial involving 199 participants revealed that the administration of tocilizumab within 6 h of symptom onset in patients with STEMI resulted in a notable enhancement in myocardial salvage index, as measured by cardiac magnetic resonance imaging after 3 to 7 days [29]. This improvement was observed in comparison to the placebo group and suggested that tocilizumab could help in myocardial salvage, although no significant impact on the final infarct size was observed.

Other IL-6 inhibitors, sarilumab and ziltivekimab, have also been shown to decrease inflammation biomarkers, which are established cardiovascular risk factors [30,31]. However, more trials are needed to assess their safety and better predict cardiovascular outcomes.

## 3. Bispecific Antibodies (BsAbs)

After the advances in monoclonal antibodies, bispecific antibodies (BsAbs) have been established as revolutionizing therapies in the field of cancer immunotherapy [45]. They are recombinant molecules that exhibit the ability to concurrently bind two distinct targets through the integration of various binding domains of desired monoclonal antibodies into a single entity. BsAbs have also been implicated as promising therapeutic agents in CVDs in several studies.

One of the limitations of using hematopoietic stem cell (HSC) therapy in cardiac repair is the restricted migratory capacity of the stem cells to the target injured myocardial tissue. To address this restriction, Lee et al. conducted a study to investigate the potential of employing BsAbs to selectively target human CD34+ stem cells to infarcted cardiac tissue [46]. After injecting post-infarction mice with HSC pre-armed with a BsAb that recognizes both CD45 present on the stem cells and myosin light chain expressed by infarcted myocardium, enhancements in myocardial function were noted within 5 weeks. A similar recent study designed a BsAb by coupling anti-CD34 and an anti-cardiac myosin heavy chain to simultaneously target circulating CD34-positive cells and injured cardiac cells in a mouse model. Results also showed that the BsAb could reduce myocardial scarring and boost cardiac recovery [47]. However, the main disadvantage is that the targeted biomarkers are only expressed during myocardial injury.

**Figure 1:**
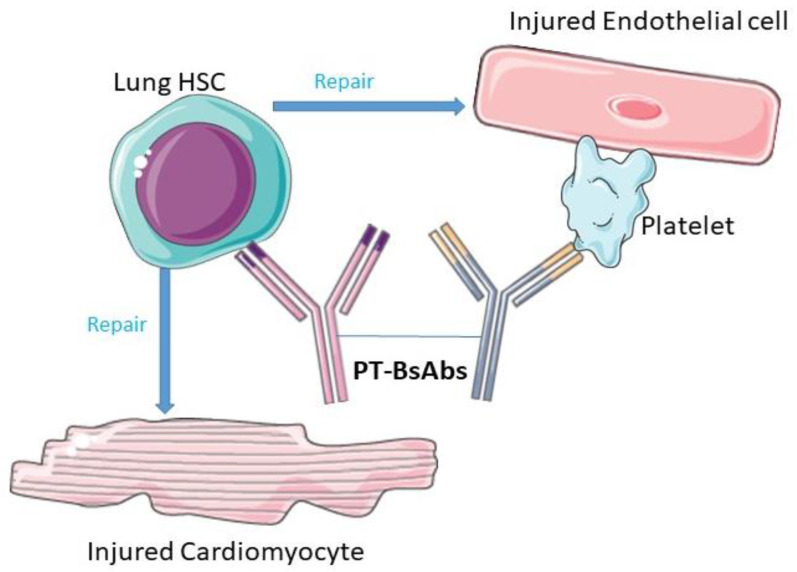
Therapeutic concept of PT-BsAb role in heart repair. Parts of the figure were drawn by using pictures from Servier Medical Art (http://smart.servier.com/, accessed on 14 December 2023), licensed under a Creative Commons Attribution 3.0 Unported License (https://creativecommons.org/licenses/by/3.0/, accessed on 14 December 2023).

In another recent study, researchers endeavored to recruit HSCs from the lungs to injured myocardial cells through inhalation therapy. The engineered BsAbs were specifically designed to target the CD34-positive HSC and CD42b receptors found on platelets, both of which are abundant in the lungs [48]. After inhalation, platelet-targeting BsAbs (PT-BsAb) could direct lung HSCs toward the damaged myocardial cells due to the inherent homing property of platelets. This novel therapy was safe and effective in increasing angio-myogenesis in the mouse model. This therapeutic concept is schematized in Figure 1.

As for the realm of vascular injuries, researchers have also attempted to enhance the regenerative vascular capacity by developing a BsAb that specifically targets two key components: the soluble platelet collagen receptor glycoprotein VI (GPVI) and the CD133 surface antigen found on endothelial progenitor cells (EPC) [49]. The bifunctional protein, GPVI-CD133, resulted in an enhancement in EPC recruitment both in pig vessels in vitro and in mice in vivo, leading to a subsequent augmentation in the regeneration of vascular lesions in the murine models.

## 4. Regulatory T-Cell Based Therapies

Regulatory T cells (Tregs) have been shown to play a key role in atherosclerosis inhibition [50]. After Webster et al. succeeded in expanding Tregs by administering interleukin (IL)-2 along with IL-2 mAb to mice [51], preclinical studies have used the IL-2/anti-IL-2 mAb treatment to expand Tregs and have demonstrated their efficacy in suppressing both the development of atherosclerosis as well as the advancement of developed atherosclerosis [52]. In mice with HF, increasing Tregs could also attenuate HF progression and decrease left and right ventricular hypertrophy and dysfunction [53]. An ongoing placebo-controlled phase I/II clinical trial aims to investigate the role of low-dose recombinant IL-2 in increasing Tregs, as well as its safety and efficacy in the setting of patients with ischemic heart disease and ACS [54]. The results of this trial may provide a promising therapeutic agent for clinical application.

## 5. Nucleic-Acid-Based Therapy

Gene therapy can be tremendously advantageous as it offers the possibility of editing and repairing disease-causing genes via novel and advanced tools such as the Clustered Regularly Interspaced Short Palindromic Repeats (CRISPR)/CRISPR-associated protein-9 (Cas9) or by introducing genes and nucleic acid molecules. To facilitate the delivery of therapeutic RNA or DNA molecules into host cells, various vectors can be used. Plasmids are among the first vectors employed in gene therapy and have demonstrated a high degree of safety, while exhibiting a low level of immunogenicity [55]. Extensive research has also been conducted on viral vectors over the course of several decades, including adenovirus (Ad), adeno-associated virus (AAV), and lentivirus [56]. To date, adenoviruses and AAVs are the viral vectors employed in cardiac clinical trials. Other methods of administration of such therapeutic agents also include direct delivery via injections. We review targeted RNA agents and focus on the advancements in this field.

### 5.1. Non-Coding RNA-Based Therapy

This therapeutic approach encompasses the use of non-coding RNA-based compounds. Specifically, their aim involves the imitation or suppression of DNA or RNA function in governing biological processes. Various technologies are employed for these methodologies, including aptamers, antisense oligonucleotides (ASOs), and RNA interference (RNAi), which is a biological process that involves the inhibition of specific genes or gene products. RNAi therapies include small interfering RNAs (siRNAs) and microRNA (miRNA) mimics or attenuation.

#### 5.1.1. Antisense Oligonucleotides

ASOs are a class of modified single-stranded oligonucleotides that can target RNAs via their sequence-specific pattern, thus affecting their function. Mipomersen is an ASO drug that has been approved by the FDA for the treatment of HoFH, a genetic condition characterized by extremely high LDL-c levels, which increases the risk for CVD [57]. After the administration of mipomersen as a subcutaneous injection, it selectively targets apoB-100 mRNA, leading to its degradation. The decrease in the apoB-100 level leads to the ultimate decrease in LDL-c levels. However, due to the risk of adverse events such as elevated liver transaminases, its use is still limited [57].

Volanesorsen is another ASO drug that has been approved by the European Medicines Agency (EMA) for the treatment of Familial Chylomicronemia Syndrome (FCS), a rare genetic condition characterized by extremely elevated levels of triglycerides. Volanesorsen works by specifically targeting and inhibiting the expression of Apolipoprotein C-III (ApoC-III), a key protein involved in triglyceride regulation. This precision-targeted approach is typically reserved for patients with FCS who have not responded to conventional therapies, given the rarity and complexity of the disorder [58]. In a pooled analysis of RCTs in patients with severe hypertriglyceridemia, volanesorsen was found to be associated with a significant decrease in triglycerides, very low-density lipoprotein, and non-high-density lipoprotein cholesterol compared to placebo [59]. Thrombocytopenia is the main adverse effect of volanesorsen use. Therefore, the platelet count in patients using volanesorsen should be monitored weekly and kept >140,000/μL [58].

Casimersen, golodirsen, viltolarsen, and eteplirsen are other ASO drugs that have been approved for the treatment of Duchenne muscular dystrophy (DMD). These ASOs are designed to bind to specific regions of the DMD RNA, causing exon skipping during the splicing process [60]. This alteration in RNA splicing helps restore the correct reading frame in dystrophin protein production. Thus, these therapies enable the synthesis of a partially functional dystrophin protein.

Pelacarsen is an ASO drug that is still under study. Pelacarsen acts by targeting apolipoprotein(a) mRNA. A recent phase II trial in patients with CVD proves that pelacarsen directly decreases lipoprotein(a) (Lp(a)) cholesterol and mildly decreases LDL-c levels [61]. Ongoing and future studies might provide more evidence of its efficacy (NCT05900141, NCT05646381, NCT05305664, NCT04023552).

#### 5.1.2. siRNA Therapy

siRNAs are a class of exogenous RNA molecules that can be used to selectively silence or downregulate the expression of specific genes.

Inclisiran is an approved synthetic siRNA-based therapeutic agent that closely mimics the activity of naturally occurring miRNA [62]. The mechanism of action of this drug involves specifically targeting and inhibiting the production of proprotein convertase subtilisin/kexin type 9 (PCSK9), resulting in lowered LDL cholesterol levels. It is intended for use in patients with primary hypercholesterolemia or mixed dyslipidemia who have not achieved adequate control through maximal statin dosage, with or without other lipid-lowering therapies (LLT) [62,63]. For patients who cannot tolerate statins or have medical reasons preventing their use, inclisiran can be used, either with or without other LLTs. What sets inclisiran apart is its dosing schedule, as it is administered as a twice-yearly subcutaneous injection, providing a convenient option for individuals who need long-term cholesterol management [62,63]. Currently, inclisiran (Leqvio) is given in a single-dose prefilled syringe (284 mg) initially, then after 3 months, and then every 6 months. The shelf life of the medication is 3 years, and no required post-injection-related safety monitoring is needed.

Olpasiran is a siRNA drug that inhibits the translation of Lp(a) mRNA in liver cells. During the phase I clinical trial, olpasiran exhibited a dose-dependent reduction in Lp(a) levels [64]. Administering a single dose of 9 mg or higher was well tolerated and resulted in a reduction of over 90% in the Lp(a) concentration in individuals with elevated levels, and this effect lasted for a duration of 3 to 6 months. Similarly, in the phase II OCEAN[a]-DOSE trial, olpasiran resulted in a significant and sustained decrease in Lp(a) levels in individuals with atherosclerotic CVD [65].

SLN-360 is also a siRNA that disrupts the production of Lp(a), and it is currently still under clinical development (NCT05537571, NCT04606602). SLN-360 has undergone a phase I clinical trial and has demonstrated its safety as well as its dose-dependent reduction in Lp-a levels [66]. Another siRNA drug candidate that targets Lp(a) levels, LY3819469, is likewise currently in the clinical development phase. The ongoing clinical trial aims to assess the pharmacokinetics and pharmacodynamics of LY3819469, as well as to evaluate its safety and tolerability (NCT04914546).

Zilebesiran is yet another siRNA potential therapeutic agent for the management of hypertension. Zilebesiran inhibits hepatic angiotensinogen mRNA, which in turn results in a decreased production of angiotensinogen, therefore theoretically reducing blood pressure [67]. A recent phase I trial in hypertensive patients assessed the safety and efficacy of zilebesiran as well as its pharmacokinetic properties [68]. The study showed that the administration of single subcutaneous doses of zilebesiran demonstrated dose-proportional reductions in both serum angiotensinogen levels and blood pressure. These reductions were sustained for up to 24 weeks. The most frequently observed adverse events were mild and temporary injection-site reactions. Future and ongoing phase II trials will better evaluate the efficacy of zilebesiran (NCT05103332).

#### 5.1.3. miRNA Therapy

miRNA is a class of endogenous short non-coding RNAs that function as post-transcriptional moderators of gene expression [69]. They exert their regulatory effects by facilitating the degradation or decay of coding mRNAs, hence modulating protein synthesis, and affecting various body functions.

Evidence has shown that miRNAs regulate cardiomyocyte proliferation and regeneration [70]. Although using antisense oligonucleotides (ASOs) to inhibit these suppressive miRNAs has a limited role in promoting cardiac regeneration therapeutically, several research groups have reported positive outcomes through administering pro-proliferative miRNAs. By using AAV9 vectors, a sustained expression of exogenous miRNAs within the heart can be achieved. In MI mouse models, the introduction of miR-199a or miR-590a [71], miR-294 from the miR-302 superfamily [72], and miR-19a/19b from the miR-17~92 cluster [73], all delivered using AAV vectors, resulted in cardiac regeneration and the restoration of cardiac function.

However, sustained expression of pro-proliferative miRNAs from AAV vectors presents potential challenges. Firstly, there is no controlled regulation of miRNA expression since the vectors used can persist indefinitely. And secondly, within the transduced cells, there is a generation of both the intended miRNA strand and the miRNA originating from the complementary strand. This production can result in unintended adverse events over time, as demonstrated in recent experiments using miR-199a in pigs, which resulted in severe arrhythmias [74]. Transient administration of miRNA mimics seems promising in addressing these challenges. Single intracardiac doses of miR-199a-3p or miR-590-3p mimics injected into the heart of mice could persist for a minimum of 12 days, triggering cardiac repair and recovery [75].

As for angiogenesis, miRNA-92a, which blocks angiogenesis when overexpressed, serves as a potential therapeutic target [76]. In MI mouse models, inhibiting miRNA-92a could enhance vascularization [76]. Similar findings were demonstrated in a large animal model of pigs where this miRNA could protect against ischemia/reperfusion injury [77]. Furthermore, research has shown that targeting miRNA-92A could serve as a suitable therapeutic approach for addressing diabetes-associated cardiac microvascular dysfunction [78].

MRG-110 and CDR132L are miRNA-based therapies approved for testing in humans (NCT03603431, NCT04045405). The first study in humans using MRG-110, a synthetic agent that targets miRNA-92a, demonstrated that administering MRG-110 in healthy adults leads to a decrease in miR-92a levels and subsequent gene target de-repression in human peripheral blood cells [79]. The synthetic agent CDR132L inhibits miRNA-132, which plays a role in pathological myocardial remodeling by inhibiting genes such as SERCA2a. After preclinical studies showed that CD132L could restore SERCA2a function and prevent adverse cardiomyocyte remodeling [80], Taubel et al. completed the first human trial using this molecule. The phase Ib trial demonstrated CDR132L safety and efficacy in reducing miRNA-132 and in improving cardiac function by decreasing heart fibrosis [81].

Table 3 summarizes the main RNA-targeted therapies studied.

## 6. T-Cell Immunotherapy

**Figure 2:**
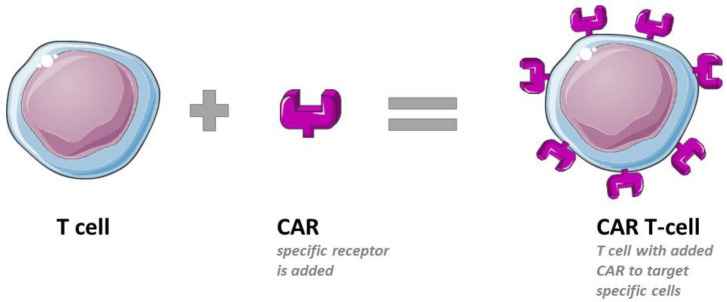
Therapeutic concept of Chimeric Antigen Receptor (CAR) T-cell therapy. Parts of the figure were drawn by using pictures from Servier Medical Art (http://smart.servier.com/, accessed on 14 December 2023), licensed under a Creative Commons Attribution 3.0 Unported License (https://creativecommons.org/licenses/by/3.0/, accessed on 14 December 2023).

Chimeric Antigen Receptor (CAR) T-cell therapy is a process in which a patient’s T-cells are genetically modified to carry a CAR antigen, as illustrated in Figure 2. This modification occurs outside the patient’s body through cell expansion, after which the cells are reintroduced into the patient [82]. In 2019, a groundbreaking approach utilized chimeric antigen receptor (CAR-T) cells to specifically target and eliminate cardiac fibroblasts expressing either ovalbumin peptide (OVA) or fibroblast activation protein alpha (FAP), both of which are prevalent in activated fibroblasts [83]. The introduction of CD8+ T cells with a chimeric antigen receptor against those antigens significantly reduced cardiac fibrosis and restored cardiac function in injured mice.

Although this approach was promising, it had some limitations. It required exogenous T cell modification and had limited control over the long-term persistence of modified CAR-T cells. A recent study conducted by the same research group has tackled these challenges by using lipid nanoparticles (LNPs) to deliver an mRNA construct, allowing for the endogenous transformation of T cells into FAP-targeting CAR-Ts (FAPCAR) [84]. This innovative method, involving the in vivo generation of CAR T cells, led to a reduction in fibrosis and the restoration of cardiac function following injury.

## 7. Nanoparticles for Drug Delivery

Nanoparticles (NPs) constitute a mode of drug delivery that aims to increase drug bioavailability, stability, and safety [85]. It is possible through the use of NPs to deliver the drug directly to the intended location by utilizing surface properties that enable targeted delivery. Optimally, a NP drug vehicle should possess the following characteristics: biodegradability, biocompatibility, stability, minimal or no immunogenicity, ability to undergo surface changes, ease of functionality, and the capacity to deliver and release encapsulated drugs at specific cellular targets [86]. NPs may be conjugated to targeted ligands such as small molecules, peptides, proteins, and antibodies that seek out cellular receptors to achieve directed drug delivery [87]. Furthermore, encapsulation within the NPs protects against unwanted alterations and degradation of the drug [88].

**Figure 3:**
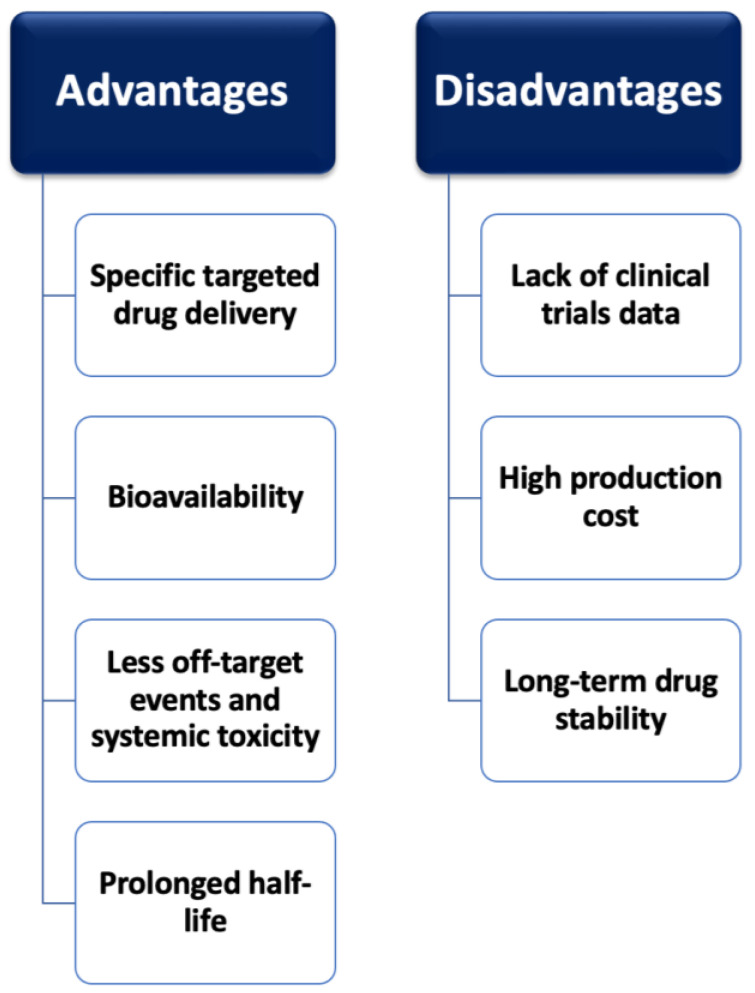
Advantages and disadvantages of nanoparticle use for targeted drug delivery.

Nanotechnology has been applied to a far greater extent in cancer therapy than in CVD. Despite this, its utility in CVD is of immense importance as its possible therapeutic benefits are linked to the role of the cardiovascular system in blood circulation, perfusion, and thus organ function [87,89,90]. The application of this mode of treatment delivery has been applied to combat various cardiovascular diseases [88]. Figure 3 presents the advantages and limitations of NP use for targeted CVD therapy.

The most common cause of heart failure is myocardial infarction, and thus a targeted approach to the deleterious effects subsequent to myocardial ischemia is crucial to improving functionality and limiting injury to the myocardium following such an event [91]. Drug delivery systems (DDS) equipped with ligands capable of binding to cell-specific receptors or moieties that are overexpressed in pathophysiological settings mostly utilize enhanced permeability and retention (EPR)-mediated passive targeting to penetrate the tissue [92]. This passively targeted modality is promoted by the increased vascular permeability and reduced microvascular flow post-MI. Ligands such as sugars, peptides, folic acid, and antibodies are used to enhance the duration of stay of the drug and its uptake by cells. For instance, it is evident through experimentation on rats that NP-carried pitivastatin serves a protective role against reperfusion injury through the phosphatidylinositol 3-kinase protein kinase B (PI3K/Akt)-mediated pathway. This limits myocardial damage, apoptosis, and inflammation following acute myocardial infarction (AMI), all features that are absent when pitivastatin is given on its own [93]. Similarly, another study showed that irbesartan containing NP limited reperfusion injury and thus infarct size through a peroxisome proliferator-activated receptor (PPAR)γ agonistic effect and attenuation of the inflammatory response mediated via monocytes [94]. These effects were also lacking in controls. Furthermore, damage subdued following an AMI is believed to be in part due to the increased production of reactive oxygen species that influence myocardial remodeling. A major contributor to macrophage superoxide free radical production is NADPH oxidase 2 (NOX2). When NOX2 is inhibited via microRNA NP and small interfering RNA NP, there is an evident improvement in myocardial function as well as a reduction in infarct size in rat models [91,95].

Smart drug delivery can also be accomplished through active targeting, which involves directing the DDS to the desired location through biological components such as overexpressed receptors on the target cells [92]. Alternatively, external magnetic fields can be used to guide iron-containing carriers in the body. Active targeting NPs have been described in MI, utilizing fibrin as a target of conjugated moieties. Fibrin is increased in the infarcted zone of the heart, a feature that can be used to increase drug delivery. This was shown in the case of thymosin beta-4-encapsulated NPs, whose concentrations were increased in the infarcted area and resulted in enhanced angiogenesis, cardiac function, and survival [96]. Another example is that of angiotensin II type 1 receptor (AT1R) ligand incorporation into NP, which is increased in ischemic myocardial tissue. The targeting of AT1R with microRNA inhibitor-loaded NP has shown decreased cellular apoptosis and infarct size [97]. Also, platelet nano-vesicles embedded on the surface of stem cells have been shown to be retained to a greater degree within ischemic and damaged zones of the heart as well as in injured vessels. This may be attributed to platelet surface molecule adhesive properties [98].

Another interesting application of this delivery system is in T-cell-targeted lipid NPs that encapsulate messenger RNA. These NPs transform T lymphocytes transiently into anti-fibrotic CAR-T cells in vivo that reduce fibrosis and improve myocardial function in mice with heart failure [84]. NP technology extends to the innate immune system, where cardiac macrophage-targeting miRNA-21-encapsulated NPs induced a switch from a pro-inflammatory phenotype to a reparative one in an injured myocardium. This immunomodulation results in enhanced angiogenesis, reduced hypertrophy, cellular apoptosis, and fibrosis [99].

In the field of arrhythmia, an innovative ablation method uses NPs containing a cardiac-targeting peptide and photosensitizer to which laser illumination is imposed, and it induces localized, myocyte-specific ablation with 85% efficiency. This method limits avoidable collateral damage, as exemplified by intact fibroblasts near ablation. Furthermore, this targeted way of reverting into a sinus rhythm provides a means to ablation that is safer and averts some of the complications set by the more classical modes [100]. Additionally, several antiarrhythmic drugs have been coupled to NPs and have shown to be of therapeutic benefit, including liposomal amiodarone-loaded NPs, which show enhanced ischemic/perfused myocardial site selectivity and efficacy. Consequently, this has reduced mortality from lethal arrhythmias while avoiding the negative hemodynamic effects of amiodarone use in such patients [101,102].

## 8. Conclusions

In conclusion, targeted therapies represent groundbreaking and promising treatments in the field of CVD. Such advancements have paved the way for precision medicine approaches, holding great promise for a new era of tailored therapy in patients with CVDs. Unfortunately, it remains mandatory to overcome the possible disadvantages and limitations of such modalities. For instance, although mAbs, BsAbs, and CAR-T therapy offer highly specific therapeutic agents with no off-target events, they require a complex preparation procedure and are extremely high in cost [103]. The disadvantages of CAR-T therapy also include possible life-threatening toxicities such as cytokine release syndrome, which mandates the need to engineer less toxic therapy forms [104]. Gene-targeted therapies, on the other hand, are easy to prepare but can lead to several off-target events [103]. Newer therapeutic methods using NP formulations for efficient drug delivery into the heart tissue offer a very promising targeted therapy potential (Figure 3). However, clinical translation is required to study their full effects in the human body.

The expansion of precision medicine through future and ongoing research will help us overcome such difficulties, ultimately improving the approach to patient care. It is expected that targeted therapies will play a significant role in providing better patient outcomes and reduced disease burden due to their synergistic treatment effects. This includes the use of therapeutic antibodies that simultaneously target multiple pathological mechanisms implicated in CVDs, such as inflammation, oxidative stress, and endothelial dysfunction. Moreover, advancements in drug delivery technologies, such as the use of nanoparticles, will also affect the future of CVD therapies. From novel drug delivery systems capable of targeted drug delivery to specific cardiac tissues or cell types to sophisticated diagnostic tools that enable real-time monitoring of treatment response and disease progression, such innovations will lead to more personalized medical care in the field of CVD.

## Figures and Tables

**Table 1:** Monoclonal Antibody Targets in CVD therapies.

Monoclonal Antibody Targets	Drugs	Trial References
PSCK9-targeted mAbs	Evolocumab	[10]
	Alirocumab	[11]
	Bococizumab	[12]
ANGPTL3 Inhibitors	Evinacumab	[13,14,15]
GP IIb/IIIa Inhibitors	Abciximab	[16,17,18,19]
	Eptifibatide	[20]
	Tirofiban	[21]
IL-1 Inhibitors	Anakinra	[22,23,24]
	Canakinumab	[25,26]
	Rilonacept	[27]
IL-6 Inhibitors	Tocilizumab	[28,29]
	Sarilumab	[30]
	Ziltivekimab	[31]

**Table 2:** IL-1 antagonists.

IL-1 Antagonist	Half-Life	Clinical Trial	Intervention	Results
Anakinra	4–6 h	Phase II	100 mg/day subcutaneous anakinra vs. placebo for 2 weeks in 10 STEMI patients	Significant improvement in the left ventricular end-systolic and end-diastolic volume index. Similar statistically significant differences in cardiac index changes among the groups. No significant difference in left ventricle ejection fraction among the 2 groups [23].
		Phase II	100 mg anakinra once per day, 100 mg anakinra twice daily, or placebo for 2 weeks in 99 STEMI patients	Significant reduction in inflammation, death, heart failure incidence, death, and hospitalization for heart failure in anakinra groups. No discernible impact on left ventricle function and ejection fraction [22].
		Phase II/III	100 mg anakinra for 12 weeks or anakinra for 2 weeks followed by placebo for 10 weeks vs. placebo for 12 weeks in 60 patients with decompensated systolic HF	Improved peak aerobic exercise capacity, patient perceptions of dyspnea on exertion (DOE), and rating of perceived exertion (RPE). No improvement in peak oxygen consumption (Vo2) and ventilatory efficiency (VE/Vco2 slope) [24].
Canakinumab	26 days	Phase III	Three dosages (50 mg, 150 mg, 300 mg) vs. placebo, administered every three months in a total of 10,061 patients with previous MI high-CRP level of 2 mg/L or more with a median follow-up of 3.7 years	Significant reduction in high-sensitivity CRP levels, significant reduction in nonfatal MI, nonfatal stroke, cardiovascular death, and urgent revascularization-requiring unstable angina (150 mg group) [25].
		Phase II	Canakinumab subcutaneous dose of 150 mg for 12 months vs. placebo in 38 patients with symptomatic peripheral artery disease	No alteration in atherosclerotic plaque progression in superficial femoral artery; improved maximal and pain-free walking distance after 3 months of treatment with canakinumab vs. placebo [26].
Rilonacept	26 days	Phase III	Rilonacept as a loading dose of 320 mg followed by maintenance doses of 160 mg once weekly vs. placebo were given over 12 weeks in 86 patients with recurrent pericarditis symptoms and systemic inflammation, evidenced by high CRP-levels	Significant reduction in pericarditis recurrence and pericarditis symptoms in recurrent episodes in the rilonacept patient group compared to placebo [27].

**Table 3:** RNA-based therapy.

Therapeutic Approach	Target	Drug/Agent	Trials	Results
Non-coding RNA: ASO	ApoB-100	Mipomersen	Approved	Decreased apoB-100 mRNA, resulting in decreased LDL-c levels in patients with homozygous familial hypercholesterolemia [57]
Non-coding RNA: ASO	ApoC-III	Volanesorsen	Approved	Inhibition of ApoC-III expression and reduction in triglycerides in patients with Familial Chylomicronemia Syndrome [58]
Non-coding RNA: ASO	Specific regions of the DMD RNA	Casimersen, Golodirsen, Viltolarsen, and Eteplirsen	Approved	Synthesis of a partially functional dystrophin protein by exon skipping during the splicing process [60]
Non-coding RNA: ASO	Apolipoprotein(a) mRNA	Pelacarsen	Phase II Trial	Pelacarsen decreases lipoprotein(a) [61]
Non-coding RNA: siRNA	PCSK9	Inclisiran	Approved	Inhibition of PCSK9 expression with lowered LDL cholesterol levels [62]
Non-coding RNA: siRNA	Lp(a)	Olpasiran	Phase I Trial, Phase II OCEAN[a]-DOSE Trial	Safety demonstrated; dose-dependent reduction in Lp(a) levels [64]; sustained decrease in Lp(a) levels [65]
Non-coding RNA: siRNA	Lp(a)	SLN-360	Phase I Trial	Safety demonstrated; dose-dependent reduction in Lp(a) levels [66]
Non-coding RNA: siRNA	Lp(a)	LY3819469	Ongoing Phase 1 Trial	No results yet published (NCT04914546)
Non-coding RNA: siRNA	Angiotensinogen mRNA	Zilebesiran	Phase I Trial	Safety demonstrated; dose-dependent reduction in angiotensinogen levels, and decrease in blood pressure [68]
Non-coding RNA: miRNA	miR-199a, miR-590a, miR-294, and miR-19a/19b	miR-199a, miR-590a, miR-294, and miR-19a/19b introduced via AAV vectors	Animal models	Cardiac regeneration and the restoration of cardiac function [71,72,73]
Non-coding RNA: miRNA	miR-199a, miR-590-3p	miR-199a and miR-590-3p mimics	Animal models	Cardiac repair and recovery [75]
Non-coding RNA: miRNA	miR-92a	MRG-110	Phase I Trial	Safety demonstrated; decrease in miR-92a levels, potential angiogenic therapeutic benefit [79]
Non-coding RNA: miRNA	miR-132	CDR132L	Phase I Trial	Safety demonstrated; decrease in miR-132 levels, improved cardiac function by decreasing heart fibrosis [81]
